# Crystal structure of 15,16-ep­oxy-7β,9α-di­hydroxy­labdane-13(16),14-dien-6-one

**DOI:** 10.1107/S2056989015011214

**Published:** 2015-06-13

**Authors:** Vikram Dev Singh, Kamni, Musarat Amina, Nawal Al-Musayeib, Sumati Anthal, Rajni Kant

**Affiliations:** a Faculty of Sciences, Shri Mata Vaishno Devi University, Katra 182 320, Jammu, J & K, India; b Department of Pharmacognosy, College of Pharmacy, King Saud University, Riyadh 11451, Saudi Arabia; c Post-Graduate Department of Physics & Electronics, University of Jammu, Jammu Tawi 180 006, India

**Keywords:** crystal structure, 15,16-epoxy-7β,9α-dihydroxylabdane-13(16),14-dien-6-one, otostegiafruticosa, biological activity, hydrogen bonding

## Abstract

In the title mol­ecule, C_20_H_30_O_4_, both cyclo­hexane rings adopt chair conformations. In the crystal, mol­ecules are connected by O—H⋯O hydrogen bonds forming chains along [100]. In addtion, an intra­molecular O—H⋯O hydrogen bond forms an *S*(5) ring.

## Related literature   

For background to the title compound, see: Al-Musayeib *et al.* (2000 ▸); Shaw (1985 ▸). For its biological activities, see: Mossa *et al.* (2000 ▸); Kidane *et al.* (2013 ▸). For the synthesis and spectroscopic data, see: Savona *et al.* (1976 ▸,1977 ▸); Hon *et al.* (1993 ▸).
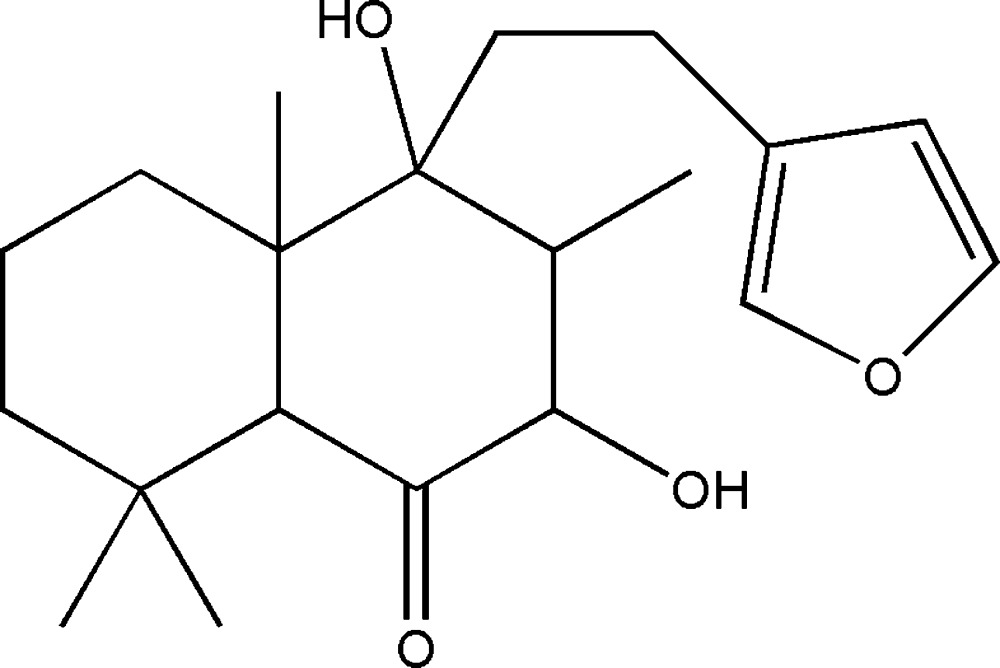


## Experimental   

### Crystal data  

C_20_H_30_O_4_
*M* *_r_* = 334.44
Orthorhombic, 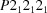

*a* = 8.5757 (7) Å
*b* = 9.2957 (8) Å
*c* = 22.994 (2) Å
*V* = 1833.0 (3) Å^3^
*Z* = 4
Mo *K*α radiation
μ = 0.08 mm^−1^
*T* = 293 K
0.30 × 0.20 × 0.20 mm

### Data collection  

Oxford Diffraction Xcalibur Sapphire3 diffractometer
Absorption correction: multi-scan (*CrysAlis PRO*; Oxford Diffraction, 2010 ▸) *T*_min_ = 0.865, *T*_max_ = 1.000
5318 measured reflections
3554 independent reflections
2457 reflections with *I* > 2σ(*I*)
*R*_int_ = 0.021

### Refinement  

*R*[*F* ^2^ > 2σ(*F* ^2^)] = 0.052
*wR*(*F* ^2^) = 0.115
*S* = 1.02
3554 reflections
226 parameters
H atoms treated by a mixture of independent and constrained refinement
Δρ_max_ = 0.12 e Å^−3^
Δρ_min_ = −0.14 e Å^−3^

### 

Data collection: *CrysAlis PRO* (Oxford Diffraction, 2010 ▸); cell refinement: *CrysAlis PRO*; data reduction: *CrysAlis PRO* (Oxford Diffraction, 2010 ▸); program(s) used to solve structure: *SHELXS97* (Sheldrick, 2008 ▸); program(s) used to refine structure: *SHELXL97* (Sheldrick, 2008 ▸); molecular graphics: *ORTEP-3 for Windows* (Farrugia, 2012 ▸); software used to prepare material for publication: *PLATON* (Spek, 2009 ▸).

## Supplementary Material

Crystal structure: contains datablock(s) I, New_Global_Publ_Block. DOI: 10.1107/S2056989015011214/lh5767sup1.cif

Structure factors: contains datablock(s) I. DOI: 10.1107/S2056989015011214/lh5767Isup2.hkl

**Fig. 1.**
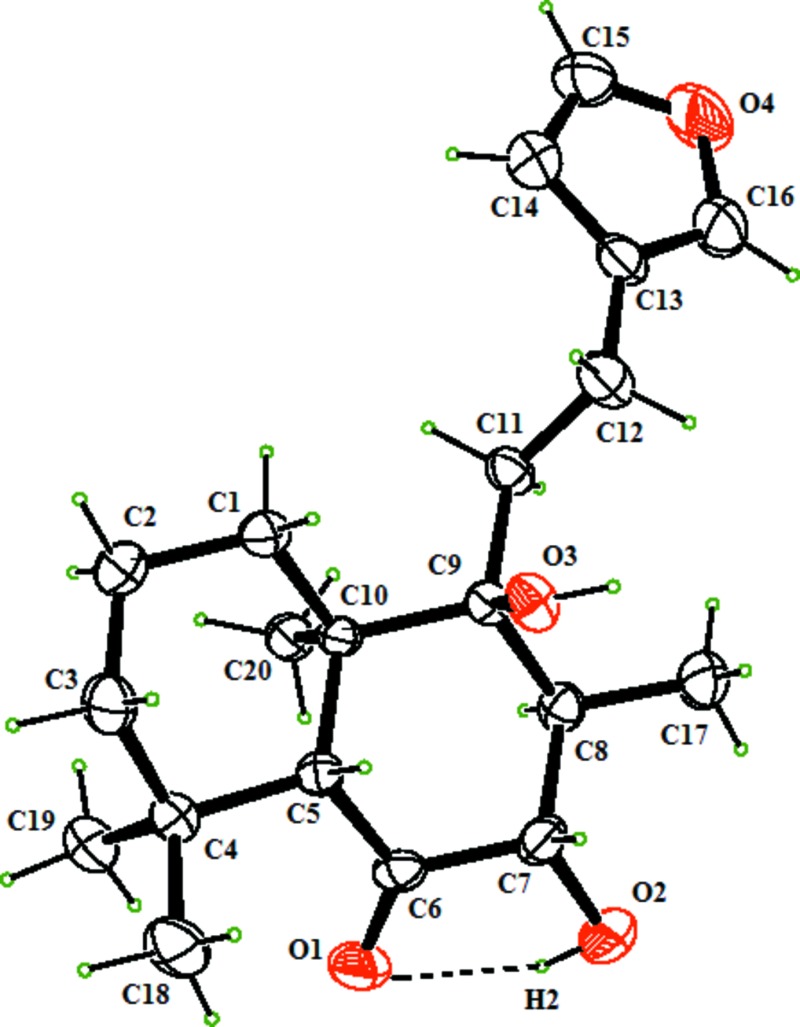
The molecular structure of the title compound with ellipsoids drawn at the 40% probability level. H atoms are shown as small spheres of arbitrary radii and the dashed line indicates an intramolecular hydrogen bond.

**Fig. 2.**
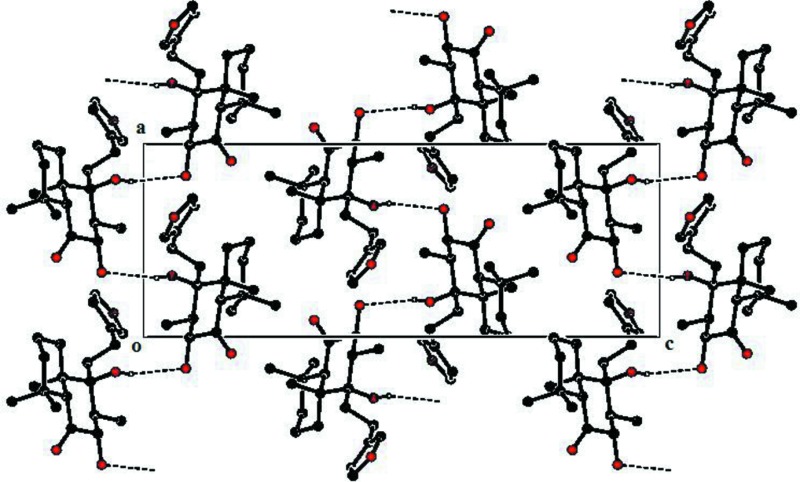
The packing arrangement of molecules viewed along the b axis. Hydrogen bonds are shown as dashed lines.

. DOI: 10.1107/S2056989015011214/lh5767fig1.tif
The mol­ecular structure of the title compound with ellipsoids drawn at the 40% probability level. H atoms are shown as small spheres of arbitrary radii and the dashed line indicates an intra­molecular hydrogen bond.

b . DOI: 10.1107/S2056989015011214/lh5767fig2.tif
The packing arrangement of mol­ecules viewed along the *b* axis. Hydrogen bonds are shown as dashed lines.

CCDC reference: 1405794

Additional supporting information:  crystallographic information; 3D view; checkCIF report

## Figures and Tables

**Table 1:** Hydrogen-bond geometry (, )

*D*H*A*	*D*H	H*A*	*D* *A*	*D*H*A*
O2H2O1	0.88(3)	2.06(4)	2.628(3)	122(3)
O3H3O2^i^	0.82	2.46	3.203(3)	151

Symmetry code: (i) 
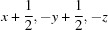
.

## supplementary crystallographic information

### S1. Comment

In a continuation of our investigations on Otostegiafruticosa (Al-Musayeib *et al.*, 2000) we report herein the isolation of Labdanediterpene, 15,16-epoxy-7β,9α-dihydroxylabdane- 13 (16),14-dien-6-one, from aerial parts of O. fruticosa. The structure of the isolate was established by spectral analysis followed by single crystal X-ray diffraction studies. The genus Otostegia (Labiatae) comprises 20 species (Shaw 1985), of which Otostegiafruticosa Forssk (Briq) is the only one found in Saudi Arabia. The plant of O. fruticosa is usually an erect, branched and straggly shrub with white flowers, up to 1.25 m in height. In Saudi Arabia, the plant grows on the rocky hills along the Jeddah-Taif road and Abha, where it is locally named Hewaymid and traditionally used as a remedy for sun-stroke (Mossa *et al.*, 2000), and as mosquito repellent (Kidane *et al.*, 2013). The molecular structure of the title compound (I) is shown in Fig. 1. The bond distances are in the normal ranges. The presence of the double bond C6═O1 is confirmed by the distance of 1.213 (3) Å. The torsion angle about the C11—C12 bond is 178.7 (2)°, indicating a *trans* conformation. Both cyclohexane rings adopts *chair* conformations. For cyclohexane ring (C5–C10), the best mirror plane passes through atoms C9 and C6 and the best two fold rotational axis bisects the C7—C10 bond with asymmetry parameters: [ΔC_s_(C9)=0.86 and ΔC_2_(C7—C8)=0.64] and in the case of ring (C1—C5/C10) the best mirror plane passes through the atoms C2 and C5 and the best two fold rotation axis bisects the C2—C3 bond with asymmetry parameters: [ΔC_s_(C2)=2.42 and ΔC_2_(C2—C3)=1.85]. There is an O—H···O intramolecular hydrogen bond between the hydroxyl groups containing atoms O2 and O1 *via* H2 which results in the formation of a pseudo five membered ring comprising of atoms O1/C6/C7/O2/H2 with S(5) graph-set motif. In the crystal, molecules are connected via O—H···O hydrogen bonds, forming chains along [100] (Fig. 2).

### S2. Experimental

Isolated areial parts of O. fruticosa were collected in the pre-flowering stage, in April, near the city of Abha, located in the Southern region of Saudi Arabia. A Voucher specimen has been deposited in the herbarium of Research Center for Medicinal, Aromatic and Poisonous Plants of College of Pharmacy, King Saud university. Air-dried and powdered aerial parts (500g) of O. fruticosa were exhaustively percolated with neutral, acetic acid-free, ethyl acetate. The solvent was evaporated and the residue was partitioned between acetonitrileand n-hexane, pre-saturated with each other. The combined hexane phases were back-washed with 100 ml of acetonitrile and combined acetonitrile phases were evaporated *in vacuo* to leave a greenish oily residue. A portion of acetonitrile fraction obtained above (5 g) was subjected to flash chromatography on a column (40 *x* 2.5 cm) of a silica gel and elution was carried in increasing polarity with n-hexane, 10% ether in hexane, 15% ether in hexane, 20% ether in hexane, and ether. Fractions got eluted in 20% ether in hexane were pooled, concentrated and residue on crystallization from n-hexane yielded LabdanediterpeneI. The title compound was obtained as fine needles, m.p 369.2 - 370.2K (from n-hexane). The molecular formula was established as C_20_H_30_O_4_ by EI mass spectrum and elemental analysis. The fragments at m/*z* 81 and 95 in the mass spectrum were indicative of presence of β-monosubstituted furan ring (Savona *et al.*, 1977;1977). IR spectrum showed the presence of hydroxyl group (3520–3428 cm-1), α, β-unsaturated ketone (1695 cm-1) and an aromatic olefinic (1575 cm-1). The 1H NMR spectrum of I was consistent with a typical β-monosubstituted furan ring, as it contained two α-furan protons with resonance at δ 7.36 (brs, H-14) and δ 7.42 (brs, H-16) and one furan proton with a resonances δ 6.27 (brs, H-14). The 1H and 13 C NMR data contained resonances attributable to three tertiary methyl singlets (δ_H_0.96, 1.29, 0.88; δ_C_32.5, 22.2, 18.0), one secondary methyl group (δ_H_1.25, d, J=6.5 Hz; δ_C_12.3), five methylene, six methines, one of which is oxygen-bearing (δ_C_77.1) and three of which were attributed to the β-substituted furan ring, and five quaternary carbon signals, including a ketonic carbonyl (δ_C_211.9) and a tertiary hydroxyl-bearing carbon atom (δ_C_77.4). The other physical and spectroscopy data is in good agreement with the literature (Hon *et al.*, 1993).

### S3. Refinement

H2 attached to O2 was located from a difference Fourier map and refined isotropically. The remaining H atoms were positioned geometrically and refined using a riding model, with C—H = 0.93–0.98 Å, O2—H2 = 0.82Å and with *U*_iso_(H) = 1.2*U*_eq_(C) or *U*_iso_(H) = 1.5*U*_eq_(C_methyl_,O).

### Crystal data

**Table:**

C_20_H_30_O_4_	*F*(000) = 728
*M**_r_* = 334.44	*D*_x_ = 1.212 Mg m^−^^3^
Orthorhombic, *P*2_1_2_1_2_1_	Mo *K*α radiation, λ = 0.71073 Å
Hall symbol: P 2ac 2ab	θ = 4.2–27.3°
*a* = 8.5757 (7) Å	µ = 0.08 mm^−^^1^
*b* = 9.2957 (8) Å	*T* = 293 K
*c* = 22.994 (2) Å	Block, white
*V* = 1833.0 (3) Å^3^	0.30 × 0.20 × 0.20 mm
*Z* = 4	

### Data collection

**Table:**

Oxford Diffraction Xcalibur Sapphire3 diffractometer	3554 independent reflections
Radiation source: fine-focus sealed tube	2457 reflections with *I* > 2σ(*I*)
Graphite monochromator	*R*_int_ = 0.021
Detector resolution: 16.1049 pixels mm^-1^	θ_max_ = 26.0°, θ_min_ = 3.5°
ω scans	*h* = −10→6
Absorption correction: multi-scan (*CrysAlis PRO*; Oxford Diffraction, 2010)	*k* = −11→9
*T*_min_ = 0.865, *T*_max_ = 1.000	*l* = −14→28
5318 measured reflections	

### Refinement

**Table:**

Refinement on *F*^2^	Secondary atom site location: difference Fourier map
Least-squares matrix: full	Hydrogen site location: inferred from neighbouring sites
*R*[*F*^2^ > 2σ(*F*^2^)] = 0.052	H atoms treated by a mixture of independent and constrained refinement
*wR*(*F*^2^) = 0.115	*w* = 1/[σ^2^(*F*_o_^2^) + (0.0448*P*)^2^ + 0.067*P*] where *P* = (*F*_o_^2^ + 2*F*_c_^2^)/3
*S* = 1.02	(Δ/σ)_max_ = 0.001
3554 reflections	Δρ_max_ = 0.12 e Å^−^^3^
226 parameters	Δρ_min_ = −0.14 e Å^−^^3^
0 restraints	Extinction correction: *SHELXL97* (Sheldrick, 2008), Fc^*^=kFc[1+0.001xFc^2^λ^3^/sin(2θ)]^-1/4^
Primary atom site location: structure-invariant direct methods	Extinction coefficient: 0.0048 (14)

### Special details

**Table:**

Experimental. *CrysAlis PRO*, Oxford Diffraction Ltd., Version 1.171.34.40 (release 27–08-2010 CrysAlis171. NET) (compiled Aug 27 2010,11:50:40) Empirical absorption correction using spherical harmonics, implemented in SCALE3 ABSPACK scaling algorithm.
Geometry. All e.s.d.'s (except the e.s.d. in the dihedral angle between two l.s. planes) are estimated using the full covariance matrix. The cell e.s.d.'s are taken into account individually in the estimation of e.s.d.'s in distances, angles and torsion angles; correlations between e.s.d.'s in cell parameters are only used when they are defined by crystal symmetry. An approximate (isotropic) treatment of cell e.s.d.'s is used for estimating e.s.d.'s involving l.s. planes.
Refinement. Refinement of *F*^2^ against ALL reflections. The weighted *R*-factor *wR* and goodness of fit *S* are based on *F*^2^, conventional *R*-factors *R* are based on *F*, with *F* set to zero for negative *F*^2^. The threshold expression of *F*^2^ > σ(*F*^2^) is used only for calculating *R*-factors(gt) *etc*. and is not relevant to the choice of reflections for refinement. *R*-factors based on *F*^2^ are statistically about twice as large as those based on *F*, and *R*- factors based on ALL data will be even larger.

### Fractional atomic coordinates and isotropic or equivalent isotropic displacement parameters (Å^2^)

**Table:**

	*x*	*y*	*z*	*U*_iso_*/*U*_eq_	
O3	0.31780 (19)	0.20597 (18)	0.05469 (7)	0.0475 (5)	
H3	0.3066	0.2523	0.0246	0.071*	
C9	0.2680 (3)	0.2912 (3)	0.10343 (10)	0.0346 (5)	
C10	0.2913 (2)	0.1911 (3)	0.15784 (10)	0.0342 (6)	
C5	0.1913 (3)	0.0511 (3)	0.14935 (10)	0.0359 (6)	
H5	0.2247	0.0130	0.1116	0.043*	
C1	0.4627 (3)	0.1443 (3)	0.16192 (12)	0.0477 (7)	
H1A	0.4966	0.1127	0.1238	0.057*	
H1B	0.5251	0.2274	0.1724	0.057*	
C6	0.0239 (3)	0.0936 (3)	0.14021 (11)	0.0412 (6)	
O2	−0.1636 (2)	0.2232 (3)	0.08150 (12)	0.0757 (7)	
O1	−0.0871 (2)	0.0563 (2)	0.16925 (8)	0.0650 (6)	
C8	0.0939 (3)	0.3308 (3)	0.09553 (11)	0.0422 (6)	
H8	0.0592	0.3799	0.1309	0.051*	
C11	0.3672 (3)	0.4308 (3)	0.10900 (10)	0.0436 (6)	
H11A	0.2981	0.5095	0.1192	0.052*	
H11B	0.4401	0.4184	0.1408	0.052*	
C12	0.4588 (3)	0.4737 (3)	0.05478 (12)	0.0598 (8)	
H12A	0.5309	0.3971	0.0449	0.072*	
H12B	0.3871	0.4854	0.0225	0.072*	
C17	0.0631 (3)	0.4306 (3)	0.04419 (13)	0.0634 (8)	
H17A	0.1023	0.3874	0.0092	0.095*	
H17B	0.1147	0.5209	0.0506	0.095*	
H17C	−0.0471	0.4464	0.0404	0.095*	
C4	0.2190 (3)	−0.0753 (3)	0.19275 (11)	0.0488 (7)	
C7	−0.0025 (3)	0.1927 (3)	0.08904 (12)	0.0487 (7)	
H7	0.0339	0.1429	0.0540	0.058*	
C20	0.2425 (3)	0.2737 (3)	0.21285 (10)	0.0457 (7)	
H20A	0.1320	0.2644	0.2184	0.068*	
H20B	0.2688	0.3735	0.2085	0.068*	
H20C	0.2961	0.2348	0.2459	0.068*	
C2	0.4949 (3)	0.0249 (3)	0.20529 (14)	0.0648 (9)	
H2A	0.6042	−0.0017	0.2034	0.078*	
H2B	0.4733	0.0592	0.2443	0.078*	
O4	0.6221 (3)	0.8402 (3)	0.05881 (12)	0.1051 (9)	
C3	0.3952 (3)	−0.1058 (3)	0.19269 (14)	0.0638 (8)	
H3A	0.4174	−0.1790	0.2216	0.077*	
H3B	0.4243	−0.1442	0.1550	0.077*	
C13	0.5481 (3)	0.6103 (3)	0.06312 (12)	0.0541 (8)	
C18	0.1353 (4)	−0.2094 (3)	0.16993 (14)	0.0689 (9)	
H18A	0.1656	−0.2266	0.1303	0.103*	
H18B	0.0246	−0.1948	0.1718	0.103*	
H18C	0.1632	−0.2909	0.1934	0.103*	
C19	0.1641 (4)	−0.0495 (3)	0.25525 (11)	0.0653 (9)	
H19A	0.1759	−0.1363	0.2774	0.098*	
H19B	0.0563	−0.0214	0.2550	0.098*	
H19C	0.2255	0.0255	0.2725	0.098*	
C14	0.6804 (4)	0.6324 (4)	0.09808 (14)	0.0708 (9)	
H14	0.7311	0.5626	0.1200	0.085*	
C16	0.5176 (5)	0.7401 (4)	0.04063 (15)	0.0877 (12)	
H16	0.4350	0.7594	0.0156	0.105*	
C15	0.7203 (4)	0.7698 (4)	0.09435 (15)	0.0841 (11)	
H15	0.8043	0.8115	0.1136	0.101*	
H2	−0.195 (4)	0.203 (4)	0.1169 (13)	0.076 (12)*	

### Atomic displacement parameters (Å^2^)

**Table:**

	*U*^11^	*U*^22^	*U*^33^	*U*^12^	*U*^13^	*U*^23^
O3	0.0644 (11)	0.0427 (11)	0.0354 (9)	−0.0007 (10)	0.0092 (8)	−0.0046 (8)
C9	0.0398 (12)	0.0324 (14)	0.0317 (12)	−0.0025 (12)	0.0025 (10)	−0.0027 (10)
C10	0.0322 (11)	0.0365 (14)	0.0337 (13)	−0.0049 (11)	0.0018 (10)	0.0005 (10)
C5	0.0356 (12)	0.0343 (14)	0.0377 (13)	−0.0017 (12)	−0.0004 (11)	−0.0019 (11)
C1	0.0355 (13)	0.0496 (17)	0.0581 (17)	−0.0036 (13)	−0.0021 (12)	0.0088 (14)
C6	0.0405 (13)	0.0324 (15)	0.0509 (16)	−0.0077 (13)	−0.0044 (12)	−0.0072 (11)
O2	0.0461 (11)	0.0863 (19)	0.0947 (19)	−0.0021 (12)	−0.0218 (12)	0.0208 (14)
O1	0.0430 (10)	0.0689 (15)	0.0832 (14)	−0.0102 (11)	0.0058 (10)	0.0128 (12)
C8	0.0454 (13)	0.0374 (16)	0.0437 (15)	0.0028 (13)	−0.0043 (12)	−0.0006 (11)
C11	0.0549 (15)	0.0384 (16)	0.0375 (14)	−0.0099 (14)	0.0024 (12)	−0.0008 (12)
C12	0.077 (2)	0.055 (2)	0.0471 (16)	−0.0151 (17)	0.0081 (15)	0.0007 (14)
C17	0.0685 (18)	0.054 (2)	0.068 (2)	−0.0020 (18)	−0.0158 (15)	0.0139 (15)
C4	0.0489 (14)	0.0418 (18)	0.0556 (17)	−0.0050 (14)	−0.0046 (13)	0.0104 (13)
C7	0.0411 (14)	0.0544 (19)	0.0507 (16)	−0.0001 (14)	−0.0141 (12)	−0.0001 (13)
C20	0.0518 (14)	0.0462 (17)	0.0390 (14)	−0.0087 (14)	−0.0015 (12)	−0.0012 (12)
C2	0.0417 (14)	0.073 (2)	0.079 (2)	0.0017 (16)	−0.0102 (16)	0.0243 (17)
O4	0.134 (2)	0.0734 (18)	0.1079 (19)	−0.0477 (18)	−0.0138 (19)	0.0282 (16)
C3	0.0555 (16)	0.051 (2)	0.085 (2)	0.0089 (16)	−0.0046 (16)	0.0230 (16)
C13	0.0673 (18)	0.0552 (19)	0.0396 (15)	−0.0193 (16)	0.0063 (14)	0.0062 (14)
C18	0.078 (2)	0.0350 (18)	0.094 (2)	−0.0063 (17)	−0.0047 (18)	0.0069 (16)
C19	0.0691 (18)	0.071 (2)	0.0562 (19)	−0.0155 (19)	0.0009 (16)	0.0186 (16)
C14	0.0682 (19)	0.074 (3)	0.070 (2)	−0.011 (2)	−0.0080 (17)	0.0168 (18)
C16	0.103 (3)	0.075 (3)	0.085 (3)	−0.037 (2)	−0.030 (2)	0.032 (2)
C15	0.085 (2)	0.090 (3)	0.077 (2)	−0.043 (2)	−0.010 (2)	0.008 (2)

### Geometric parameters (Å, º)

**Table:**

O3—C9	1.438 (3)	C17—H17B	0.9600
O3—H3	0.8200	C17—H17C	0.9600
C9—C8	1.548 (3)	C4—C18	1.531 (4)
C9—C11	1.556 (3)	C4—C19	1.531 (4)
C9—C10	1.572 (3)	C4—C3	1.537 (3)
C10—C1	1.536 (3)	C7—H7	0.9800
C10—C20	1.538 (3)	C20—H20A	0.9600
C10—C5	1.571 (3)	C20—H20B	0.9600
C5—C6	1.504 (3)	C20—H20C	0.9600
C5—C4	1.560 (3)	C2—C3	1.514 (4)
C5—H5	0.9800	C2—H2A	0.9700
C1—C2	1.518 (4)	C2—H2B	0.9700
C1—H1A	0.9700	O4—C15	1.344 (4)
C1—H1B	0.9700	O4—C16	1.358 (4)
C6—O1	1.213 (3)	C3—H3A	0.9700
C6—C7	1.511 (4)	C3—H3B	0.9700
O2—C7	1.421 (3)	C13—C16	1.339 (4)
O2—H2	0.88 (3)	C13—C14	1.406 (4)
C8—C17	1.525 (4)	C18—H18A	0.9600
C8—C7	1.534 (4)	C18—H18B	0.9600
C8—H8	0.9800	C18—H18C	0.9600
C11—C12	1.527 (3)	C19—H19A	0.9600
C11—H11A	0.9700	C19—H19B	0.9600
C11—H11B	0.9700	C19—H19C	0.9600
C12—C13	1.495 (4)	C14—C15	1.325 (4)
C12—H12A	0.9700	C14—H14	0.9300
C12—H12B	0.9700	C16—H16	0.9300
C17—H17A	0.9600	C15—H15	0.9300
			
C9—O3—H3	109.5	C18—C4—C3	108.1 (2)
O3—C9—C8	109.01 (18)	C19—C4—C3	109.4 (2)
O3—C9—C11	111.19 (17)	C18—C4—C5	108.8 (2)
C8—C9—C11	109.78 (19)	C19—C4—C5	115.8 (2)
O3—C9—C10	104.86 (18)	C3—C4—C5	106.8 (2)
C8—C9—C10	110.88 (18)	O2—C7—C6	111.2 (2)
C11—C9—C10	111.01 (18)	O2—C7—C8	111.7 (2)
C1—C10—C20	110.6 (2)	C6—C7—C8	110.69 (19)
C1—C10—C5	107.2 (2)	O2—C7—H7	107.7
C20—C10—C5	111.54 (18)	C6—C7—H7	107.7
C1—C10—C9	109.75 (18)	C8—C7—H7	107.7
C20—C10—C9	108.94 (19)	C10—C20—H20A	109.5
C5—C10—C9	108.79 (17)	C10—C20—H20B	109.5
C6—C5—C4	115.7 (2)	H20A—C20—H20B	109.5
C6—C5—C10	108.70 (19)	C10—C20—H20C	109.5
C4—C5—C10	117.47 (18)	H20A—C20—H20C	109.5
C6—C5—H5	104.5	H20B—C20—H20C	109.5
C4—C5—H5	104.5	C3—C2—C1	111.0 (2)
C10—C5—H5	104.5	C3—C2—H2A	109.4
C2—C1—C10	114.9 (2)	C1—C2—H2A	109.4
C2—C1—H1A	108.5	C3—C2—H2B	109.4
C10—C1—H1A	108.5	C1—C2—H2B	109.4
C2—C1—H1B	108.5	H2A—C2—H2B	108.0
C10—C1—H1B	108.5	C15—O4—C16	105.5 (3)
H1A—C1—H1B	107.5	C2—C3—C4	114.0 (3)
O1—C6—C5	126.6 (2)	C2—C3—H3A	108.7
O1—C6—C7	119.0 (2)	C4—C3—H3A	108.7
C5—C6—C7	114.3 (2)	C2—C3—H3B	108.7
C7—O2—H2	98 (2)	C4—C3—H3B	108.7
C17—C8—C7	109.9 (2)	H3A—C3—H3B	107.6
C17—C8—C9	113.7 (2)	C16—C13—C14	104.3 (3)
C7—C8—C9	109.4 (2)	C16—C13—C12	128.0 (3)
C17—C8—H8	107.9	C14—C13—C12	127.6 (3)
C7—C8—H8	107.9	C4—C18—H18A	109.5
C9—C8—H8	107.9	C4—C18—H18B	109.5
C12—C11—C9	115.6 (2)	H18A—C18—H18B	109.5
C12—C11—H11A	108.4	C4—C18—H18C	109.5
C9—C11—H11A	108.4	H18A—C18—H18C	109.5
C12—C11—H11B	108.4	H18B—C18—H18C	109.5
C9—C11—H11B	108.4	C4—C19—H19A	109.5
H11A—C11—H11B	107.4	C4—C19—H19B	109.5
C13—C12—C11	112.4 (2)	H19A—C19—H19B	109.5
C13—C12—H12A	109.1	C4—C19—H19C	109.5
C11—C12—H12A	109.1	H19A—C19—H19C	109.5
C13—C12—H12B	109.1	H19B—C19—H19C	109.5
C11—C12—H12B	109.1	C15—C14—C13	108.2 (3)
H12A—C12—H12B	107.9	C15—C14—H14	125.9
C8—C17—H17A	109.5	C13—C14—H14	125.9
C8—C17—H17B	109.5	C13—C16—O4	111.7 (3)
H17A—C17—H17B	109.5	C13—C16—H16	124.1
C8—C17—H17C	109.5	O4—C16—H16	124.1
H17A—C17—H17C	109.5	C14—C15—O4	110.3 (3)
H17B—C17—H17C	109.5	C14—C15—H15	124.8
C18—C4—C19	107.7 (2)	O4—C15—H15	124.8
			
O3—C9—C10—C1	58.2 (2)	C10—C9—C11—C12	133.9 (2)
C8—C9—C10—C1	175.7 (2)	C9—C11—C12—C13	178.7 (2)
C11—C9—C10—C1	−62.0 (3)	C6—C5—C4—C18	60.4 (3)
O3—C9—C10—C20	179.43 (17)	C10—C5—C4—C18	−169.0 (2)
C8—C9—C10—C20	−63.1 (2)	C6—C5—C4—C19	−61.1 (3)
C11—C9—C10—C20	59.3 (2)	C10—C5—C4—C19	69.5 (3)
O3—C9—C10—C5	−58.8 (2)	C6—C5—C4—C3	176.8 (2)
C8—C9—C10—C5	58.7 (2)	C10—C5—C4—C3	−52.5 (3)
C11—C9—C10—C5	−178.96 (18)	O1—C6—C7—O2	−2.5 (3)
C1—C10—C5—C6	−175.4 (2)	C5—C6—C7—O2	177.4 (2)
C20—C10—C5—C6	63.4 (2)	O1—C6—C7—C8	122.3 (3)
C9—C10—C5—C6	−56.8 (2)	C5—C6—C7—C8	−57.8 (3)
C1—C10—C5—C4	50.9 (3)	C17—C8—C7—O2	−54.3 (3)
C20—C10—C5—C4	−70.3 (3)	C9—C8—C7—O2	−179.9 (2)
C9—C10—C5—C4	169.49 (19)	C17—C8—C7—C6	−178.8 (2)
C20—C10—C1—C2	71.2 (3)	C9—C8—C7—C6	55.6 (3)
C5—C10—C1—C2	−50.6 (3)	C10—C1—C2—C3	55.8 (3)
C9—C10—C1—C2	−168.6 (2)	C1—C2—C3—C4	−57.4 (3)
C4—C5—C6—O1	12.5 (4)	C18—C4—C3—C2	171.0 (2)
C10—C5—C6—O1	−122.2 (3)	C19—C4—C3—C2	−72.0 (3)
C4—C5—C6—C7	−167.4 (2)	C5—C4—C3—C2	54.1 (3)
C10—C5—C6—C7	57.9 (3)	C11—C12—C13—C16	−107.2 (4)
O3—C9—C8—C17	−66.3 (3)	C11—C12—C13—C14	70.4 (4)
C11—C9—C8—C17	55.7 (3)	C16—C13—C14—C15	−0.2 (4)
C10—C9—C8—C17	178.7 (2)	C12—C13—C14—C15	−178.3 (3)
O3—C9—C8—C7	57.0 (2)	C14—C13—C16—O4	0.5 (4)
C11—C9—C8—C7	179.04 (19)	C12—C13—C16—O4	178.5 (3)
C10—C9—C8—C7	−57.9 (3)	C15—O4—C16—C13	−0.5 (4)
O3—C9—C11—C12	17.6 (3)	C13—C14—C15—O4	−0.1 (4)
C8—C9—C11—C12	−103.1 (2)	C16—O4—C15—C14	0.4 (4)

### Hydrogen-bond geometry (Å, º)

**Table:**

*D*—H···*A*	*D*—H	H···*A*	*D*···*A*	*D*—H···*A*
O2—H2···O1	0.88 (3)	2.06 (4)	2.628 (3)	122 (3)
O3—H3···O2^i^	0.82	2.46	3.203 (3)	151

Symmetry code: (i) *x*+1/2, −*y*+1/2, −*z*.
