# Supplementary figures and images for: Crystal structure of 15,16-ep­oxy-7β,9α-di­hydroxy­labdane-13(16),14-dien-6-one
Source: Acta Crystallogr E Crystallogr Commun. 2015 Jun 13;71(Pt 7):o483–4. doi: 10.1107/S2056989015011214 (PMC4518982; doi:10.1107/S2056989015011214)

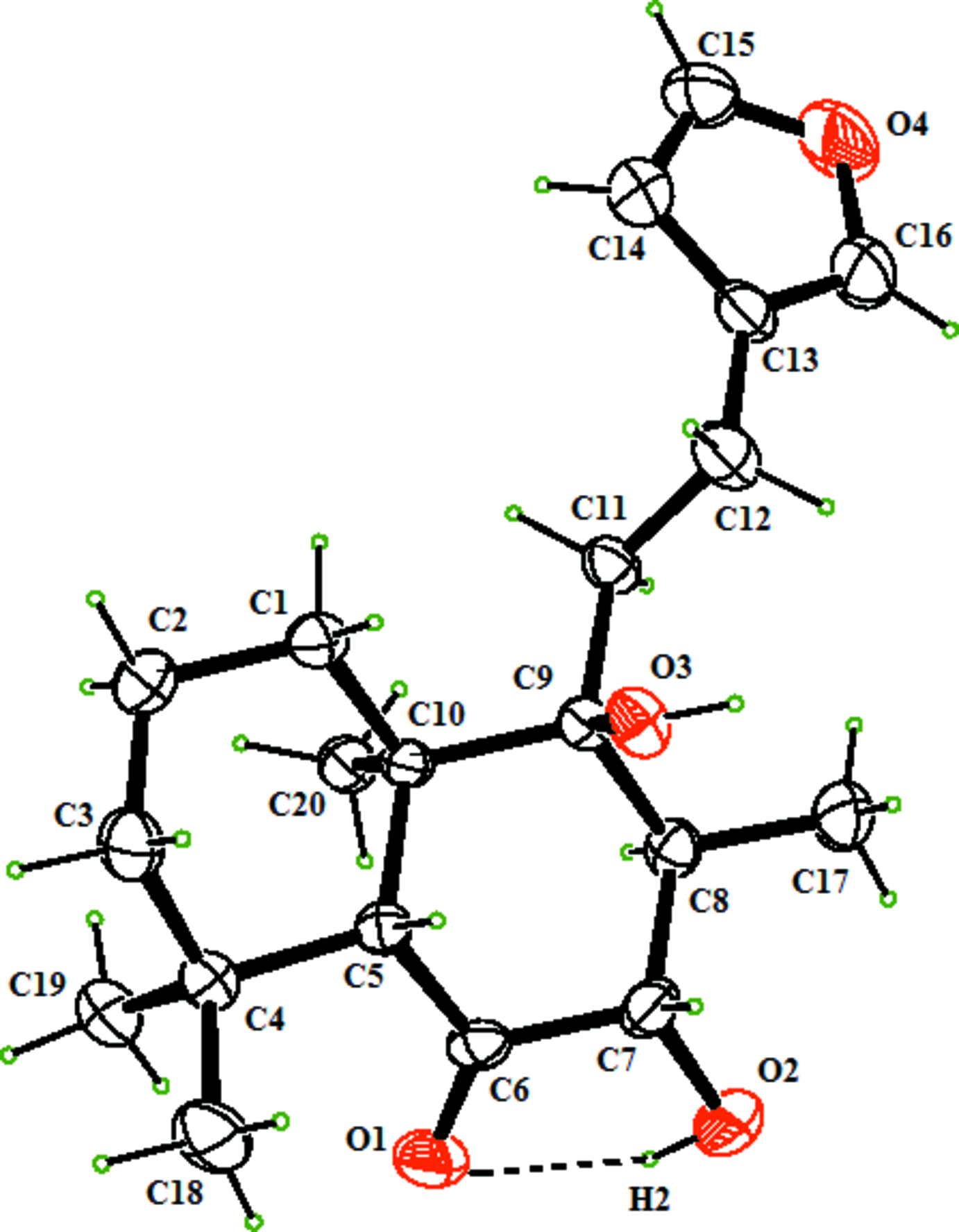

Supplement: Supplementary file 3 [file e-71-0o483-fig1.tif]

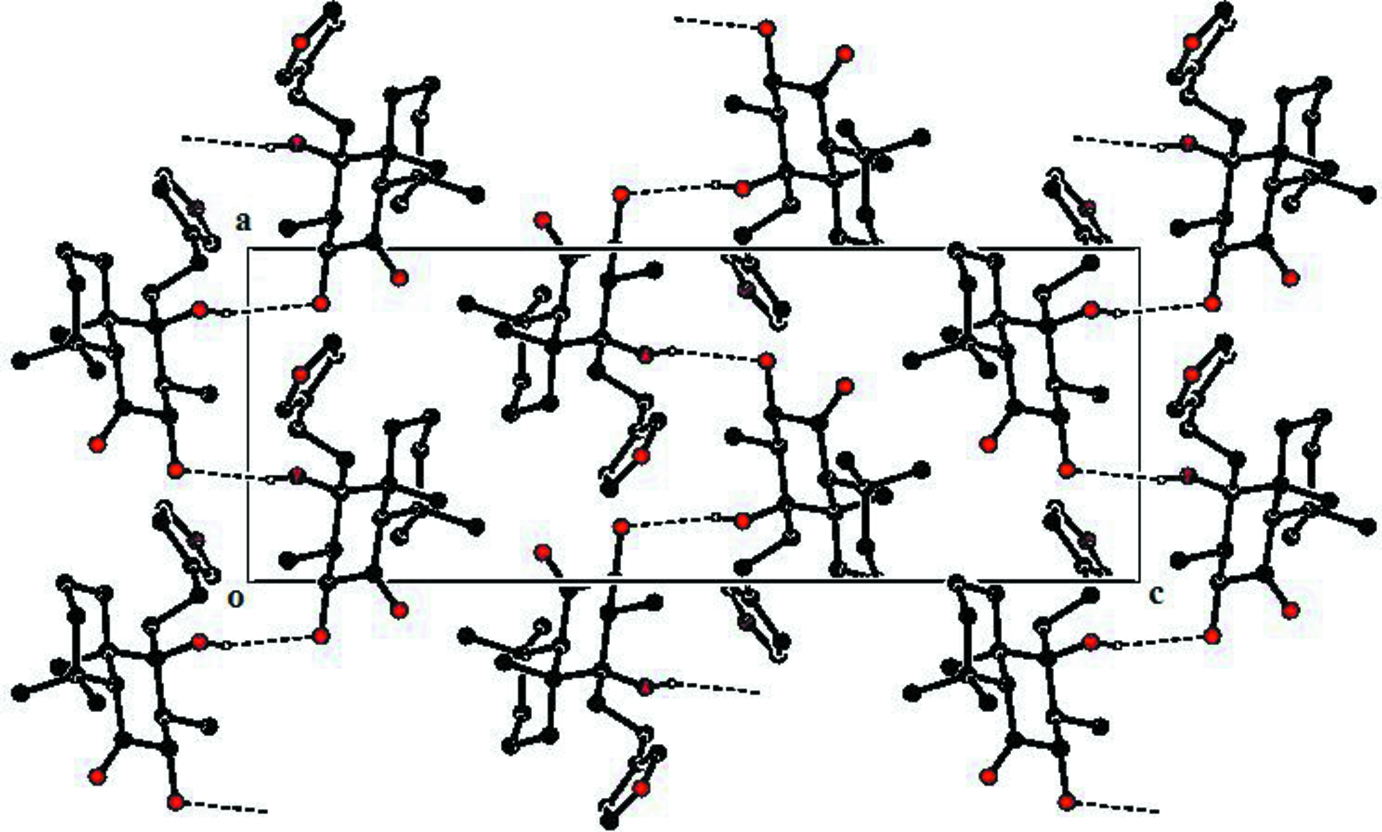

Supplement: Supplementary file 4 [file e-71-0o483-fig2.tif]
